# Association rule mining of time-based patterns in diabetes-related comorbidities on imbalanced data: a pre- and post-diagnosis study

**DOI:** 10.1186/s12911-025-03206-1

**Published:** 2025-09-29

**Authors:** Róbert Bata, Amr Sayed Ghanem, Eszter Vargáné Faludi, Ferenc Sztanek, Attila Csaba Nagy

**Affiliations:** 1 Department of Epidemiology, Faculty of Health Sciences, University of Debrecen, Debrecen, Hungary; 2 Division of Metabolic Diseases, Department of Internal Medicine, University of Debrecen Faculty of Medicine, Debrecen, Hungary

**Keywords:** Type 2 diabetes, T2DM, ICD, Comorbidities, Association rule mining, Clinical data, Imbalanced data

## Abstract

Type 2 diabetes mellitus (T2DM) is affecting over 529 million adults and anticipated to impact 1.3 billion by 2050. This disease often coexists with multiple comorbidities, which can complicate its management. These comorbidities not only increase morbidity and mortality but also challenge the effectiveness of interventions designed to manage diabetes and improve patient outcomes. We analysed imbalanced data of 25.065 patients deriving from the Clinical Centre of the University of Debrecen, Hungary. The aim of the study was to explore the prevalence and temporal patterns of comorbidities before and after the diagnosis of T2DM using Association Rule Mining (ARM) and network visualization. The initial five years following T2DM diagnosis mark a spike in newly emerging health conditions. Hypertension frequently occurs at an earlier stage, while pneumonia, eye-related disorders, and ischemic heart disease consistently appear throughout the progression of the disease. The ARM analysis showed that both acute and chronic kidney diseases, as well as respiratory disorders are common after T2DM diagnosis. Certain gender-specific trends, such as higher instances of heart failure and acute kidney injury in males, are also notable. The study highlights how ARM techniques reveal complex patterns in chronic disease management, suggesting potential pathways for targeted treatments.

**Supplementary information:**

The online version contains supplementary material available at 10.1186/s12911-025-03206-1.

## Background

Type 2 diabetes mellitus (T2DM) is a leading metabolic disorder worldwide, strongly associated with obesity [1]. All types of diabetes affect 529 million adults aged 20 to 79 years globally, the age-standardised total diabetes prevalence was 6.1% in 2021 [2]. Globally, the age-standardised prevalence of diabetes is higher in males than in females, estimated at 6.5% (95% UI 6.2–7.0) versus 5.8% (5.4–6.1), respectively [2].

Not only the high number of affected patients that is worrying, but also the rate of increase in prevalence. The prevalence of T2DM increased rapidly: in 1990, it was the eighteenth leading cause of death, but by 2017, it had moved up to seventh place [3]. It is predicted that by 2050 1.3 billion people will suffer from diabetes [2]. The proportion of patients with T2DM accounts for 90% of all types of diabetes [4].

Patients with diabetes often have other comorbidities, nearly every third patient have three or more other chronic diseases. According to the studies, the rate of patients with comorbidities doubles after 10 years [5]. According to other studies, people with diabetes’ comorbidity rate can be higher, nearly 90% [6]. The development of comorbidities are influenced by many factors e.g. sex, age, place of residence, economic status, duration of diabetes, family history of diabetes, type of diabetes, type of treatment, proteinuria, and glycaemic control [7].

Comorbid conditions associated with diabetes can be divided into two main groups: concordant and discordant diseases. The difference between the two groups is whether they have a relationship with diabetes in their pathophysiological profile or care management or not [8]. The most common concordant comorbidities are hypertension, chronic kidney disease, coronary artery disease, retinopathy, hyperlipidaemia, peripheral vascular diseases, heart failure and neuropathy; the discordant comorbidities include depression, back pain, arthritis, lung diseases and cancers [5, 8, 9].

Association rule mining (ARM) is one of the most significant techniques, in the field of data mining and knowledge discovery in databases (KDD), focusing on revealing hidden patterns in complex data environments. In the field of data mining, ARM techniques have consistently been developed by researchers of the field to get better performing algorithms [10, 11], to be able to introduce the rule mining techniques into new domains [12, 13], and to be able to unlock new capabilities for the technique [14–16]. ARM has been applied to numerous challenges in the Healthcare sector. To highlight some of them, Concaro et al. developed a method for mining temporal association rules from hybrid events and effectively integrated this method both to clinical and administrative data [17]. ARM has been employed to mine multimorbidity patterns, providing a broader understanding of the complex interplay of diseases within specific patient clusters [18–21]. It has also been applied to explore associations related to particular diseases, disorders, and symptoms, including heart disease [22], hypertension [23], borderline personality disorder [24], and to uncover symptom patterns among patients with COVID-19 [25].

ARM has been widely applied to diabetes data, primarily to uncover patterns of comorbidities and support risk assessment in various clinical contexts [26–32]. While previous studies have used ARM to analyse comorbidity patterns (or laboratory markers) in diabetes, to the best of our knowledge, this is the first study to investigate temporal comorbidity patterns in people with T2DM (ICD-10 code E11) using ARM within a framework that explicitly addresses data imbalance. We combined ARM with graph-based visualizations and adjusted ratios for temporal frequency comparisons to map, in detail, how comorbidities emerge and evolve over five-year intervals before and after T2DM diagnosis.

## Methods

### ICD-10 codes

The International Classification of Diseases, Tenth Revision (ICD-10), published by the World Health Organization (WHO), is the global standard for diagnosing and classifying diseases and health conditions. In this system, E11 denotes T2DM, with further subdivisions specifying complications; for example, E1120 indicates T2DM with renal complications. The ICD-10 employs a hierarchical structure to organize diagnoses. In our dataset, we used only the first three characters of each ICD-10 code to represent diagnostic categories, as more detailed codes would lead to overly complex and less interpretable association rules. Additionally, our analysis focused exclusively on ICD-10 codes ranging from A to N, thereby excluding codes related to pregnancy, perinatal conditions, congenital anomalies, symptoms, external causes, and other non-morbidity classifications to minimize noise and concentrate on relevant morbidities.

### Data processing

The data were assembled from the administrative records of the Clinical Centre of the University of Debrecen from 2007 up until 2021. Out of 1,028,374 patients listed in the hospital’s *source* database 65,467 patients were diagnosed with T2DM (6,3%) and were selected to the *sample* database before the data cleansing process. The assembled dataset contained patient id, age, gender, year of hospital attendance, diagnosis of T2DM, time passed before/after diagnosis of diabetes, diagnosis of comorbidities (list of diagnosis (ICD codes) made when visiting the hospital), and mortality information. For each patient, multiple records captured different hospital visits. These data were aggregated on an annual basis, grouping by patient ID and year to construct a yearly diagnostic profile for each individual. The first occurrence of the diagnosis was kept in the patient clinical history so the first diagnosis of T2DM and the proper date of the diagnosis for the comorbidities could be mapped adequately. An extra variable was calculated where the passed time was measured between the diagnosis of diabetes and the newly diagnosed disease or condition.

$$\eqalign{ & time\,\,passed\,\,before/after\,\,diagnosis\,\,of\,diabetes \cr & \, = \,\,comorbidity\,\,diagnosis\,\,date - \,diagnosis\,\,of\,E11 \cr} $$

In Supplementary Table 1. the partial database can be seen with the original occurrence of the diagnosis and the diagnosis list for the morbidities which was prepared by preserving just the first occurrence of the diagnosis in the patient health history.

It was of vital importance to have punctual diagnosis date for T2DM, because the occurrence of comorbidities and conditions were analysed in relation to it. The accuracy of the diagnosis date of T2DM was secured in three phases:

1. The duplicate occurrence of the diagnoses were removed and kept just the first occurrence.
2. Where textual health records were available for a patient (77%), and these contained a reference to the presence of diabetes with an originating date, this date was extracted using regular expressions (REGEX). If the extracted originating date preceded the diagnosis date recorded in our database, the database entry was updated (8%) to reflect the earlier date derived from the textual data.
3. Moreover, those patients with diabetes were selected who attended to the hospital preliminary to the diagnosis of T2DM without having their diabetes diagnosed.

**Fig. 1:**
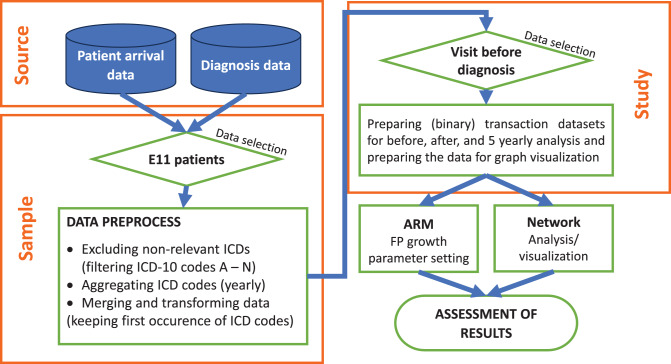
Data process this figure represents the entire process of the analysis. Initially, the database for patients with T2DM were assembled. Subsequently, data for those patients were selected who had a preliminary hospital visit without having been diagnosed with diabetes. In the end, the data were prepared and analysed using ARM and Network analysis

As a result of the preprocessing phase, patients older than 100 years were excluded as outliers, along with those who lacked prior hospital visits and the diagnosis of T2DM (ICD-10 code E11) could not be confirmed through textual health records. This reduced the sample dataset to 57,943 patients. For the final study database, we further restricted the cohort to patients who had at least one hospital visit within five years prior to their T2DM diagnosis. Consequently, the study dataset comprised 25,065 patients. (Fig. 1).

### Association rule mining (ARM)

The aim of ARM is to find patterns and combinations that both satisfy a minimal condition for prevalence and occur together far more frequently than would be anticipated under statistical independence. In this study, the transaction dataset consisted of sets of ICD codes associated with each patient. A categorical variable had been added to the transaction dataset that expresses the time passed before/after the diagnosis of T2DM. As a result, a transaction contained ICD codes for morbidities denoted with *A* for pattern and a categorical variable for time denoted with *c* for class. The categorical value labels are the following: five years before the diagnosis: *n5-n1*/0 to 4 years after diagnosis (a.d.): *0–4*/5 to 9 years a.d.: *5–9*/10 and more years a.d.:*10-*.

In the interpretation of the ARM multiple measures is evaluated in the selection of the meaningful rules. The characteristics of the dataset and the specific study objectives defined the settings for minimum thresholds and rule selection. Although the support-confidence framework for the rule selection is widely used and studied [23, 33], by using only these metrics for rule selection we might end up with several unreliable rules [34]. By incorporating the lift metric, which captures the interestingness of a rule, we can quickly and easily evaluate the quality of the mined rules [35, 36]. However, due to substantial class imbalance in our dataset, the traditional metrics of support, confidence were not sufficient for this analysis. Therefore, we adopted metrics recommended by Gu et al. [37], specifically designed to address the challenges introduced by imbalanced data. *Local support* is used to standardize the support value for the specific classes. *Lift* is not sensitive to class imbalance and is typically used to evaluate the strength of associations within a single class, indicating whether the co-occurrence of items is more (lift > 1), less (lift < 1), or about equally (lift ≈1) likely than expected by chance. In this study, *Exclusiveness* (lift) was also used to compare the relative strength of interesting rules across different classes. The exclusiveness metric for lift provides a normalized value, between 0 and 1, that is fair for all the classes. ARM can be represented as a rule in the form *A* $\to$ *c*, where *A* and *c* are disjoint item sets. *A* is the antecedent *c* is the consequent of the rule. A more detailed description of the ARM metrics, including support, confidence, lift is provided in the Supplementary Methods: Association Rule Mining Metrics.

$$Local\,\,Support\,\left( {A \to c} \right) = \frac{{support\,\left( {Ac} \right)}}{{support\left( c \right)}}$$

$$Lift \left( {A \to c} \right) = {{support\left( {Ac} \right)} \over {support\left( A \right)*support\left( c \right)}}$$

$$Exclusiveness\,\left( {Lift} \right)\left( {A \to ci} \right) = \frac{{lift\left( {A \to ci} \right)}}{{\mathop \sum \nolimits_j^{\left| c \right|} lift\left( {A \to cj} \right)}}$$

To illustrate how to interpret the association rules, consider the occurrence of *polyneuropathy in diseases classified elsewhere* during the 0–4 year timeframe. The local support of 0.076 indicates that 7.6% of people with T2DM has this condition recorded in the 0–4 year time period. The lift value of 1.77, being greater than 1, demonstrates a positive association with the discussed timeframe (0–4 years), meaning this comorbidity occurs 1.77 times more frequently than expected by chance. Notably, the exclusive lift for the 0–4 year period is relatively high at 0.452, which is given that exclusiveness lift values range between 0 and 1. This suggests that within the first five years following a T2DM diagnosis, this condition is comparatively more specific to patients with T2DM than in other time periods, highlighting a temporal concentration of this comorbidity.

Preliminary all the rules were generated with the FP-growth algorithm. The problem with using the traditional metric, support, as a uniform pruning criterion is that the same minimum support threshold cannot be effectively applied across all classes. For the most underrepresented classes, it is necessary to set a much lower support threshold (e.g., 0.001) to extract a comparable number and quality of rules to what is achieved for adequately represented classes using a higher threshold (e.g., 0.05). This results in rules of varying strength and can undermine direct comparability; by using local support, the same minimum threshold can be consistently applied across all classes to obtain rules of comparable quality. Based on a combined data-driven assessment and expert consultation, we employed a local support threshold of 0.03 to ensure statistical robustness and clinical relevance of the mined association rules (see Table 1). Local support and exclusiveness lift were calculated for the generated rules. After rule generation, we filtered for rules where the consequent was the categorical variable (timeframe) and the antecedent consisted of exactly one morbidity (e.g., a single item in the antecedent set). The rules were sorted by lift in decreasing order, highlighting the comorbidities most indicative of specific timeframes, and the top 15 rules were selected for detailed discussion. While the network graphs illustrate the 15 most highly connected morbidities overall, the association rules uncover the most notable temporal patterns within particular periods. In the manuscript, we discuss these top 15 rules ranked by lift to emphasize the most prominent associations, whereas Supplementary Table 2 provides the complete list of all rules generated at the 0.03 local support threshold. By restricting the results to the top 15, this approach also makes it easier to compare our findings with those from the other analytical methods employed.

**Table 1:** Rules by threshold

Timeframes	≥0.01	≥0.02	≥0.03	≥0.04	≥0.05
n5-n1	175	98	72	57	39
0–4	215	119	86	68	51
5–9	176	99	64	41	25
10-	58	58	36	24	17

The number of generated rules by ARM from 0.01 to 0.05 threshold. In the research we used the rules generated by the 0.03 threshold

### Network visualization

Graph networks were prepared to complete the findings of the ARM analysis. For the graph generation the same data segmentation (by timeframe) was used as for the ARM analysis. When preparing the data for the graphs for the nodes *degree centrality* and *node size* was calculated and *weight for the edges*. The *degree centrality* measures the number of connections a node has; in other words, it counts only the unique connection between the diagnosis. The diameter of the nodes (*node size*) corresponds to the number of occurrences of a particular diagnosis in the database. An *edge of weight* = 1 between two nodes represents a single subject with both diagnoses, so the edge number implies the occurrence of both diagnoses which concur the *support* of the set of the two diagnoses. Networks were generated with Gephi (version 0.10) using Force Atlas 2 layout. The nodes were sorted in descending order on the basis of degree centrality, the first 15 nodes were selected for representation, if the degree centrality was the same then the nodes with the same degree centrality were represented as well. The node size variable was used to resize the nodes proportionately in the graph representation. The edge weights were used to emphasize the strength of the relation between the two nodes.

### Statistical analysis

For the generated rules, we explored the association between gender and the occurrence of a specific disease within a given timeframe. To quantify the relationships, crude odds ratio (OR) was calculated with a 95% confidence interval (CI). These ratios were determined by comparing the odds of disease occurrence in females to those in males within the designated timeframe, using females as the reference group, males as the comparison group. To assess how the occurrence of comorbidities changed over time, we calculated adjusted ratios inspired by the method of Lee et al. [21], which treats the time period as a variable influencing disease frequency. This ratio quantifies the relative prevalence of each comorbidity across four defined timeframes: five years before the T2DM diagnosis (n5–n1), the first five years after diagnosis (0–4), and two subsequent 5-year intervals [4–8] and [9-]). It ranges from 0 to 4, where higher values indicate that a comorbidity is more concentrated in a particular period relative to its overall distribution. A ratio greater than 1 indicates a higher occurrence compared to other periods, while a ratio below 1 suggests a lower occurrence.

## Results

### Description of study data

The study database contains 25,065 patients with T2DM diagnosis. Those patients had been selected to the study database who had hospital attendance within a five-year timeframe preceding the diagnosis of T2DM, considering that morbidities occurring in this timeframe might affect the later diagnosis of T2DM. The mean age is 63.2 (SD ±13.9), 46% percent of the patients are male. The age distribution of diabetes patients is left skewed, peaking around 65–70 years (Supplementary Figure 2). The detailed dispersion and central tendency of age, patient number, gender, death of patients by the examined timeframes can be seen in Table 2.

**Table 2:** Descriptive statistics of the discussed timeframes

Diagnosis Time	Patient (n)	Patient (%)	Male (%)	Death (%)	Age (mean)	Age (SD)	Age (median)	Age (IQR)
n5-n1	25065	100	45.72	0	60.22	14.35	61	18
0–4	24893	99	45.72	9.61	63.90	13.77	65	18
5–9	9494	38	42.08	9.99	67.01	12.10	68	16
10-	2519	10	42.28	11.63	68.09	11.59	68	15

% used for percentage; IQR The interquartile range spans from the first quartile to the third quartile

Males were diagnosed at an earlier age (62.5 ± 13.1) than females (63.7 ± 14.5). The concordant diseases occurring with the highest prevalence among all the patients are hypertension (66%), hyperlipidaemia (38%), angina pectoris (33%), ischaemic heart disease (33%), diabetic retinopathy (30%), obesity (24%), chronic heart failure (22%). The discordant conditions appearing with the highest prevalence are: pneumonia (24%), spondylosis (23%) and dorsalgia (20%). The average adjusted ratios for the timeframes are as follows: n5-n1: 1.14 (SD ±0.7), 0–4: 1.33 (SD ±0.5), 5–9: 0.94 (SD ±0.5), 10-: 0.59 (SD ±0.6). By examining the adjusted ratios for the 15 most prevalent conditions (see Supplementary Table 3), all conditions show decreasing number of cases after the diagnosis of T2DM, except for pneumonia, chronic heart failure, chronic kidney disease their occurrence remain steady. Hypertension 1.13 [95% CI 1.07–1.19], ischaemic heart disease 1.14 [1.08–1.20], pneumonia 1.12 [1.06–1.19], chronic heart failure 1.18 [1.11–1.26] and atherosclerosis 1.16 [1.09–1.23] occurred with a higher likelihood in males (see Table 3). A baseline statistic for the cohort is provided in Supplementary Table 4.

**Table 3:** Top 15 most prevalent disease

Condition	Patien (n)	Patient (%)	OR (f) [95% CI]
Hypertension	16513	0.659	1.13 [1.07–1.19]
Hyperlipidaemia	9503	0.379	0.99 [0.94–1.04]
Disorders of refraction and accommodation	8699	0.347	0.76 [0.72–0.80]
Angina pectoris	8389	0.335	0.99 [0.94–1.04]
Ischemic heart disease	8164	0.326	1.14 [1.08–1.20]
Diabetic retinopathy	7457	0.298	0.81 [0.77–0.86]
Senile cataract	6003	0.239	0.68 [0.64–0.72]
Pneumonia	5985	0.239	1.12 [1.06–1.19]
Obesity	5893	0.235	0.74 [0.69–0.78]
Spondylosis	5740	0.229	0.81 [0.76–0.86]
Other disorders of eye and adnexa	5473	0.218	0.92 [0.86–0.97]
Chronic heart failure	5450	0.217	1.18 [1.11–1.26]
Dorsalgia	5100	0.203	0.84 [0.79–0.90]
Atherosclerosis	5043	0.201	1.16 [1.09–1.23]
Chronic kidney disease	4713	0.188	0.87 [0.81–0.92]

Frequency and crude odds ratios (OR) with 95% CI for selected comorbidities in patients with T2DM. n = number of patients; (%) = proportion in the cohort. ORs use females as the reference group.

### Association rules by timeframe

The aim of the rule selection is to select the most interesting rules, with a set minimum local support level, occurring in a specific timeframe. In the −5 to −1 years (n5-n1) timeframe 72 rules reached the minimum threshold. The top 15 rules (see Table 4.), with lift values ranging from 1.314–1.612, implying morbidities stronger than expected association to the timeframe (n5-n1). The topmost morbidities by lift are osteoporosis (lift: 1.612), upper respiratory infection (lift: 1.605) and excessive, frequent and irregular menstruation (1.528). Upper respiratory infection with the 0.592 exclusiveness value indicates a very high degree of uniqueness to this timeframe compared to the other timeframes and it is notable that almost all the other morbidities in the list has close to 50% exclusiveness to this timeframe out of the four timeframes. The odds relations are significantly different from 1 (lower than 1) in in the vast majority of this timeframe, which indicates lower occurrence of these morbidities in males compared to females.

**Table 4:** Results of ARM for the n5-n1 timeframe

condition	l.supp.	lift	excl. lift	OR (f) [95% CI]
Osteoporosis	0.102	1.612	0.580	0.20 [0.18–0.23]
Upper respiratory infection	0.036	1.605	0.592	0.66 [0.57–0.75]
Excessive, frequent and irregular menstruation	0.033	1.528	0.569	0.00 [0.00–0.00]
Spondylosis	0.138	1.492	0.549	0.79 [0.73–0.85]
Other soft tissue disorders, not elsewhere classified	0.091	1.472	0.452	0.80 [0.74–0.88]
Dorsalgia	0.119	1.449	0.461	0.83 [0.77–0.90]
Menopausal and other perimenopausal disorders	0.071	1.421	0.512	0.00 [0.00–0.00]
Other enthesopathies	0.044	1.405	0.508	0.67 [0.59–0.76]
Benign mammary dysplasia	0.063	1.364	0.468	0.05 [0.04–0.06]
Polyarthrosis	0.041	1.359	0.495	0.50 [0.44–0.57]
Other inflammation of vagina and vulva	0.033	1.347	0.496	0.00 [0.00–0.00]
Disorders of refraction and accommodation	0.187	1.333	0.496	0.78 [0.73–0.83]
Other intervertebral disc disorders	0.096	1.327	0.408	0.86 [0.79–0.93]
Other dorsopathies, not elsewhere classified	0.042	1.322	0.469	0.59 [0.51–0.67]
Calculus of kidney and ureter	0.046	1.314	0.468	1.05 [0.94–1.19]

Association rule mining (ARM) metrics and crude odds ratios (OR) with 95% confidence intervals (CI) for selected comorbid condition in patients with T2DM. l.supp. = local support (proportion of patients with T2DM and the condition); lift = co-occurrence relative to chance; excl. lift = specificity to T2DM on a scale from 0 to 1. ORs are calculated with female patients as the reference group.

In the 0 to 4 years (0–4) timeframe 86 rules were generated. The top 15 rules (see Table 5.), with lift values ranging from 1.401–1.766 indicating the morbidities with strong association to this timeframe. The topmost rules on the basis of the lift metric are polyneuropathy (lift: 1.766), hyperuricemia (lift: 1.581), chronic respiratory failure (lift: 1.542). On the basis of the exclusiveness metric the following morbidity diagnoses show substantial degree of specificity for this timeframe: obesity (exclusiveness (e): 0.530), hyperlipidaemia (e: 0.485), atrioventricular and left bundle-branch block (e: 0.466), polyneuropathy (e: 0.452), hyperuricemia (e: 0.435) and rheumatic tricuspid valve diseases (e: 0.388). For the most interesting rules in the 0–4 timeframe the following conditions were significantly more common in males than in females: chronic respiratory failure 1.48 [95% CI 1.35–1.63], other respiratory disorders 1.37 [1.20–1.56], other disorders of fluid, electrolyte and acid-base balance 1.12 [1.02–1.24], acute kidney injury 1.26 [1.12–1.43], chronic heart failure 1.19 [1.10–1.28], atrioventricular and left bundle-branch block 1.48 [1.28–1.71].

**Table 5:** Results of ARM for the 0–4 timeframe

antecedents	l.supp.	lift	excl. lift	OR (f) [95% CI]
Polyneuropathy in diseases classified elsewhere	0.076	1.766	0.452	0.99 [0.90–1.09]
Hyperuricemia	0.060	1.581	0.435	0.91 [0.82–1.01]
Chronic respiratory failure	0.074	1.542	0.314	1.48 [1.35–1.63]
Other disorders of fluid, electrolyte and acid-base balance	0.069	1.540	0.344	1.12 [1.02–1.24]
Other respiratory disorders	0.039	1.540	0.329	1.37 [1.20–1.56]
Chronic kidney disease	0.116	1.530	0.350	0.90 [0.83–0.97]
Acute kidney injury	0.044	1.505	0.301	1.26 [1.12–1.43]
Volume depletion	0.052	1.488	0.311	0.82 [0.73–0.92]
Other anaemias	0.094	1.486	0.351	0.93 [0.85–1.01]
Rheumatic tricuspid valve diseases	0.047	1.472	0.388	0.90 [0.80–1.02]
Hyperlipidaemia	0.225	1.466	0.485	1.05 [0.99–1.11]
Obesity	0.136	1.435	0.530	0.85 [0.79–0.91]
Chronic heart failure	0.124	1.412	0.360	1.19 [1.10–1.28]
Atrioventricular and left bundle-branch block	0.031	1.405	0.466	1.48 [1.28–1.71]
Nonrheumatic aortic valve disorders	0.042	1.401	0.368	0.99 [0.87–1.12]

Association rule mining (ARM) metrics and crude odds ratios (OR) with 95% confidence intervals (CI) for selected comorbid condition in patients with T2DM. l.supp. = local support (proportion of patients with T2DM and the condition); lift = co-occurrence relative to chance; excl. lift = specificity to T2DM on a scale from 0 to 1. ORs are calculated with female patients as the reference group.

For the 5 to 9 years [4–8] timeframe 64 rules were generated with the set threshold. The top 15 rules (see Table 6), with lift values ranging from 1.085–1.570, indicating morbidities with a stronger than expected association to the timeframe [4–8]. The topmost morbidities by lift are acute viral infection (lift: 1.570), acute kidney injury (lift: 1.527) and peripheral arterial disease (lift: 1.467). On the basis of the exclusiveness metric the following morbidities show notable degree of excess for this timeframe: other disorders of bone density and structure (e: 0.433), diverticulosis of large intestine (e: 0.374) and peripheral arterial disease (e: 0.332). Our findings indicate higher likelihood of the following diseases occur in male after the diagnosis of T2DM from 5 to 9 years: peripheral arterial disease 2.05 [95% CI 1.64–2.55], chronic respiratory failure 1.25 [1.07–1.47], other respiratory disorders 1.33 [1.07–1.66] and occlusion and stenosis of precerebral arteries, not resulting in cerebral infarction 1.44 [1.20–1.73].

**Table 6:** Results of ARM for the 5–9 timeframe

antecedents	l.supp.	lift	excl. lift	OR (f) [95% CI]
Acute viral infection	0.062	1.570	0.271	1.00 [0.84–1.18]
Acute kidney injury	0.045	1.527	0.306	1.10 [0.91–1.34]
Peripheral arterial disease	0.036	1.467	0.332	2.05 [1.64–2.55]
Volume depletion	0.050	1.431	0.299	0.88 [0.73–1.06]
Chronic respiratory failure	0.069	1.431	0.291	1.25 [1.07–1.47]
Other disorders of bone density and structure	0.033	1.413	0.433	0.30 [0.22–0.40]
Other respiratory disorders	0.035	1.389	0.297	1.33 [1.07–1.66]
Other disorders of fluid, electrolyte and acid-base balance	0.061	1.353	0.302	1.07 [0.90–1.27]
Other polyneuropathies	0.052	1.281	0.301	1.16 [0.97–1.39]
Chronic kidney disease	0.095	1.252	0.287	0.86 [0.75–0.99]
Other mental disorders	0.042	1.222	0.301	0.70 [0.57–0.86]
Diverticulosis of large intestine	0.032	1.167	0.374	0.92 [0.73–1.17]
Cystitis	0.080	1.165	0.287	0.79 [0.68–0.92]
Polyneuropathy in diseases classified elsewhere	0.049	1.142	0.293	1.09 [0.91–1.32]
Occlusion and stenosis of precerebral arteries, not resulting in cerebral infarction	0.052	1.085	0.272	1.44 [1.20–1.73]

Association rule mining (ARM) metrics and crude odds ratios (OR) with 95% confidence intervals (CI) for selected comorbid condition in patients with T2DM. l.supp. = local support (proportion of patients with T2DM and the condition); lift = co-occurrence relative to chance; excl. lift = specificity to T2DM on a scale from 0 to 1. ORs are calculated with female patients as the reference group.

For the 10 and above year timeframe [9-] timeframe 36 rules were generated. The top 15 rules (see Table 7), with lift values ranging from 0.948–2.600, indicating morbidities with stronger than expected association to the timeframe [9-]. However, the last two rules, having lift values slightly below 1, imply decreased likelihood of occurrence in this timeframe. The topmost morbidities by lift are Acute viral infection (lift: 2.600), unspecified kidney failure (lift: 2.427) and Acute kidney Injury (1.737). On the basis of the exclusiveness metric the following morbidities show notable degree of excess for this timeframe: acute viral infection (e: 0.449), unspecified kidney failure (e: 0.437) and chronic respiratory failure (lift: 0.349). The study found that, in the 10 years and above timeframe, males had a significantly higher incidence of the following conditions compared to females: acute kidney injury 1.64 [1.15–2.34], chronic respiratory failure 1.44 [1.08–1.91], lower urinary infection 1.51 [1.06–2.14], and chronic heart failure 1.75 [1.32–2.32].

**Table 7:** Results of ARM for the 10- timeframe

antecedents	l.supp.	lift	excl. lift	OR (f) [95% CI]
Acute viral infection	0.102	2.600	0.449	0.99 [0.76–1.28]
Unspecified kidney failure	0.035	2.427	0.437	1.45 [0.95–2.22]
Acute kidney injury	0.051	1.737	0.348	1.64 [1.15–2.34]
Chronic respiratory failure	0.083	1.715	0.349	1.44 [1.08–1.91]
Volume depletion	0.054	1.569	0.328	1.10 [0.78–1.56]
Other respiratory disorders	0.037	1.487	0.318	1.01 [0.67–1.54]
Other diseases of digestive system	0.031	1.450	0.319	1.21 [0.77–1.90]
Other disorders of fluid, electrolyte and acid-base balance	0.057	1.277	0.285	1.35 [0.97–1.89]
Chronic kidney disease	0.094	1.232	0.282	1.11 [0.84–1.45]
Other anaemias	0.076	1.202	0.284	0.98 [0.73–1.33]
Lower urinary infection	0.052	1.151	0.275	1.51 [1.06–2.14]
Pneumonia	0.107	1.106	0.273	1.25 [0.97–1.61]
Other polyneuropathies	0.044	1.084	0.255	0.98 [0.67–1.44]
Chronic heart failure	0.084	0.952	0.243	1.75 [1.32–2.32]
Cystitis	0.065	0.948	0.234	1.06 [0.77–1.46]

Association rule mining (ARM) metrics and crude odds ratios (OR) with 95% confidence intervals (CI) for selected comorbid condition in patients with T2DM. l.supp. = local support (proportion of patients with T2DM and the condition); lift = co-occurrence relative to chance; excl. lift = specificity to T2DM on a scale from 0 to 1. ORs are calculated with female patients as the reference group.

### Network visualization by timeframe

**Fig. 2:**
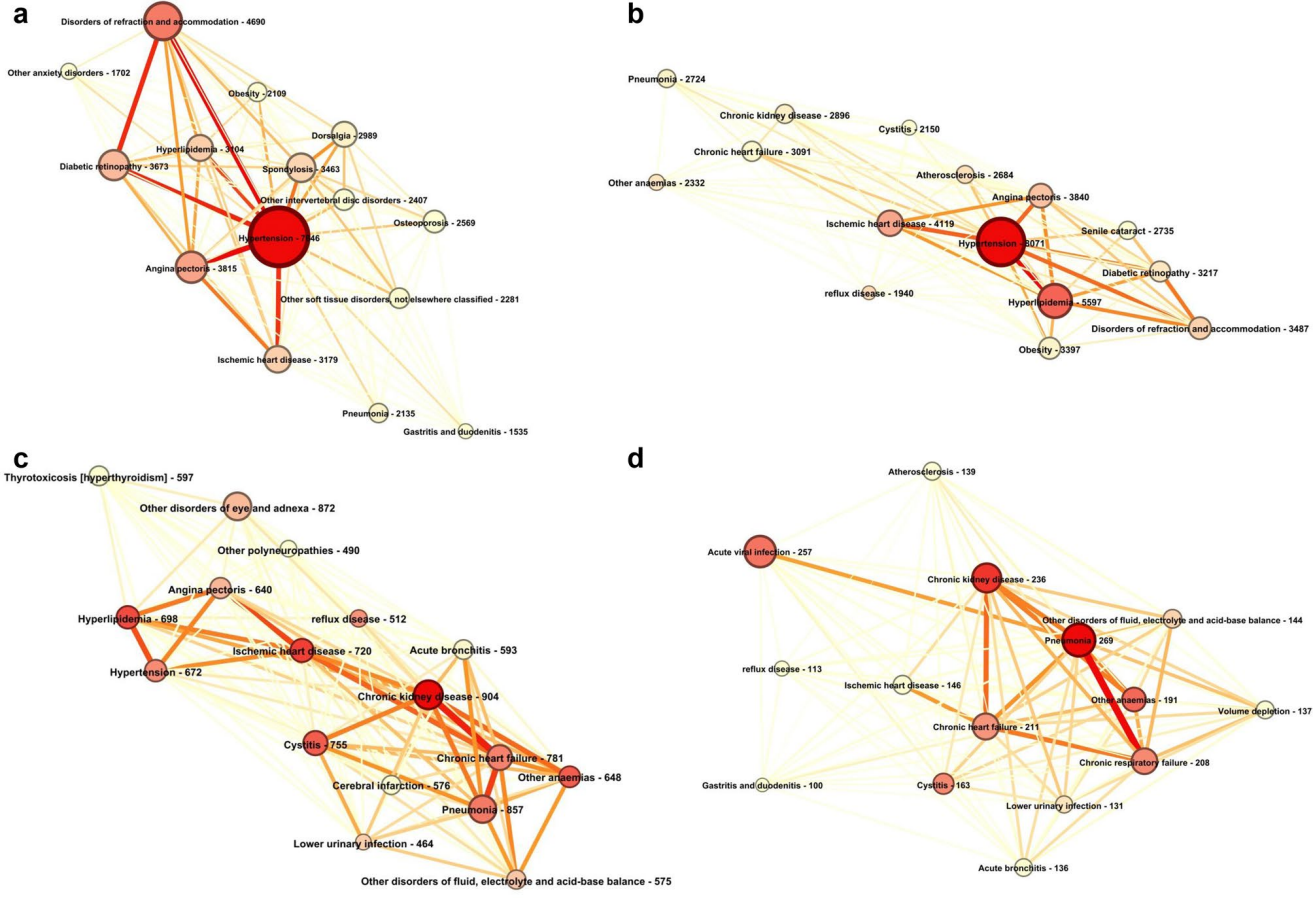
Network representation by timeframe. The first 15 diseases on the basis of degree centrality in different timeframes. (**a**) five years before T2DM diagnosis (n5-n1) sample size (n): 25.065; (**b**) 0–4 years after T2DM diagnosis, n: 24.893; (**c**) 5–9 years after T2DM diagnosis, n: 9494; (**d**) 10- years after T2DM diagnosis, n: 2519 the node size and colour indicate the occurrence of a disease, the edge colour indicates the occurrence number of two interconnected diseases. The colour indicates increasing number cooccurrences from yellow to red

Aim of the graph visualization is to represent the top 15 most prevalent diseases in a specific timeframe (see Fig. 2.). Those 15 diseases are selected which has the most connection with other diseases (degree centrality). The node size and colour indicate the occurrence of a disease; the edge colour indicate the occurrence number of two interconnected diseases. The colour indicates increasing number cooccurrences from yellow to red. In the five-year timeframe (n5-n1) preceding the diagnosis of T2DM the most prevalent conditions are hypertension (30%), disorders of refraction and accommodation (19%), and angina pectoris (15%). Hypertension is the central condition and it has high number of occurrences with the following diseases angina pectoris (10%), disorder of refraction and accommodation (10%), diabetic retinopathy (9%), and ischaemic heart disease (9%). In the first 5-year timeframe (0–4) after the diagnosis of T2DM the most prevalent condition is hypertension (32%), hyperlipidaemia (22%), and ischaemic heart disease (17%). Hypertension is the central condition and its occurrence is the highest with the following diseases disorders of lipoprotein metabolism and other lipidaemia (12%), ischaemic heart disease (9%) and angina pectoris (8%). In the 5-to-9-year timeframe [4–8] after the diagnosis of T2DM the following diseases are the most prevalent chronic kidney disease (10%), pneumonia (9%), other disorders of eye and adnexa (9%), and chronic heart failure (8%). There are more diseases taking the central role in this timeframe: chronic kidney disease, ischaemic heart disease, and hyperlipidaemia. The most frequently cooccurring conditions within this timeframe are chronic kidney disease and chronic heart failure (3%), chronic heart failure and pneumonia (2%). In the timeframe 10 years and above [9-] after the diagnosis of T2DM. There are more central conditions, and they are identical with the most prevalent diseases in the timeframe pneumonia (10%), acute viral infection (10%) and chronic kidney disease (9%). The most frequently cooccurring diseases are pneumonia and chronic respiratory failure (3%), chronic heart failure and chronic kidney disease (2%).

## Discussion

The main goal of the study was to discover the most relevant comorbidities and conditions occurring post T2DM diagnosis. ARM data mining technique was used to determine interesting patterns of items for a specific timeframe pre and post of the diagnosis of T2DM. As a supplementary tool graph were prepared to contribute the results of ARM. Instead of representing the growing number of comorbidities during the development of T2DM as it has already been discussed by a several studies [5, 9, 38, 39], the focus of our study was to identify the most relevant occurrence of certain conditions in a specific timeframe.

The first five years after being diagnosed with T2DM, including the time of diagnosis, are marked by a higher number of new diagnoses of related health conditions compared to other periods, adjusted ratio 1.33 (SD ±0.5), indicating that the occurrences are above the average ( > 1), it is in line with the findings of Pearson-Stuttard et al. [5] that in the first five years the average number of comorbidities goes up to 2.4 including that at the time of the diagnosis of T2DM a patient has 1.7 comorbidity. In the analysed data hypertension had the highest occurrence (66%) with patients with diabetes and takes the central role five years before and after the diagnosis of T2DM. With the development of the disease the occurrence of hypertension was gradually reducing the same way as Nowakowska et al. represented in their longitudinal analysis [5]. Furthermore we can confirm their findings concerning the relatively stable prevalence rates for chronic kidney disease (CKD), congenital heart disease (CHD), in case of CHD we found steady occurrence of chronic heart failure as a common cause of morbidity and mortality in CHD [40]. The constant number of renal complications align with the findings of Gregg et al. [41], that there has been a substantial reductions in complications like myocardial infarction, stroke, and amputation, while the occurrence of renal complication is more persistent. Besides CKD and CHD pneumonia also had constant occurrence among patients after the diagnosis of T2DM. Kornum et al. [42] discovered that T2DM is associated with 1.2-fold increased risk of hospitalization due to pneumonia.

The gender of the patients significantly affects the development of T2DM [43]. Interestingly, in the 5-year preceding the diagnosis of T2DM the most significant associations are predominantly more likely to occur in females. Conditions like osteoporosis, menstruation-related disorders, and menopausal disorders and vulvovaginitis are female related conditions. Kwan et al. discovered that both menopause and menstrual irregularities significantly increase the risk of diabetes [44]. Kalra et al. highlighted, that the common occurrence of vulvar pain might be indicative to diabetic neuropathy [45]. Among the most indicative rules there were certain conditions occurring in all the timeframes like respiratory failure/disorders which were more highly associated with male gender. Even though the respiratory system related diseases are discordant diseases, their number is higher among people with diabetes than in the general population reflected by the study of De Santi et al. [46] After the diagnosis of T2DM acute kidney injury (AKI) and chronic kidney disease (CKD) occur in all the timeframes among the first 15 most interesting conditions. We discovered that not by far, but AKI is more closely associated to males in the 0–4-year and 10-year timeframes and CKD is more closely correlated to the females in the 0–4-year and 5–9-year timeframes. According to the comprehensive review by Piani et al. [47] on renal diseases in patients with diabetes, while the prevalence of CKD is higher in women than men in the United States (15% vs 12%), that is consistent with our findings, the actual occurrence of renal diseases significantly varied by gender.

By comparing the relevance of specific conditions across various timeframes, we attained some interesting findings. In the 5-year timeframe preceding the diagnosis of diabetes. Some of the most class specific rules were osteoporosis and back pain (Spondylosis and Dorsalgia). As it is indicated in the article of Wongdee and Charoenphandhu [48], (pre)diabetes influences osteoporosis by increasing the bone fragility through disruptions in bone cell function and abnormal extracellular matrix structures, mainly caused by hyperglicemia and insulin resistance. The revealing article of Rinaldo et al. [49] suggests that the uncontrolled diabetes may lead to chronic back pain through mechanisms such as hyperglycemia-induced intervertebral disc degeneration. In the first five years following the diagnosis of T2DM the pronounced relevance of obesity, polyneuropathy and hyperlipidaemia is expected, all these conditions are closely linked to the progression of diabetes. However, the unexpected presence of left bundle branch block, with really high association to the timeframe (e: 0.466), among the most interesting conditions demands further investigation. As a recent study of Wittström et al. [50] introduced that patients with T2DM has a higher incidence rate of diverticular disease compared with patients without diabetes. Moreover, their results illustrated how the risk associated with T2DM may vary with the duration of the disease and called the attention to the importance of other influencing factors. We have discovered notable association of diverticular disease to the 5–9-year timeframe, while Wittström et al. found that higher risk for moderate duration (2.5–4.9 years) and decreasing risk for long duration (5+ years). These numbers might be influenced with the taken medication as [51] the article of Tseng represented that Metformin reduces the risk of diverticular disease. Acute viral infection was the most relevant condition for the 10 and above time period which supports that T2DM increases the susceptibility to severe viral infections [52].

## Strengths and limitations

To the best of our knowledge this is the first study to use ARM to analyse the occurrence of comorbidities over the course of the T2DM. The range of the analysed conditions was not restricted by using umbrella terms for the labelling of chronic conditions, so we can get more specific disease occurrences in certain timeframes with the use of the 3 long ICD codes, tough it makes the direct comparison harder to other articles where conditions were categorized. The most relevant diseases in certain timeframes were studied via different filters: in the graph presentation the most prevalent diseases are considered with the most centrally positioned diseases, while the results of the rule generation present most relevant diseases for a timeframe by the lift and the relevance of the diseases can be observed in relation to other timeframes by the exclusiveness metric. The study gained advantage from utilizing electronic health records that contain extensive data collected over several years by the Clinical Centre of Debrecen, Hungary. Due to the nature of data some limitations are implied. First the selection might be biased since a single hospital’s data was used. The institute is a secondary care provider where the more severe conditions are diagnosed not having knowledge of the happenings in the primary care service, resulting in underrepresented common diseases. After the diagnosis of diabetes patients are closely monitored in the hospital, resulting in the discovery of other comorbidities with higher rate which otherwise might remain unnoticed. Secondly, the longitudinal analysis has its own limitations. The span of the availability of data from 2007 until 2021 contributed to the unbalanced nature of the timeframes. It can be easily accepted that having a 14-year-long window for the data will result in less and less patients to represent the groups with having the diabetes diagnosis for a longer period of time. Even though we attempted to gain samples to the underrepresented timeframes by using regular expressions on the textual health records to correct the diagnosis date of T2DM where the actual (first occurrence) diagnosis dated before 2007, the sample size does not increase significantly in the underrepresented timeframes. With strict multi-criteria selection of the patients, we managed to define the date of T2DM diagnosis punctually, however it slightly contributed to the smaller sample sizes in the timeframes. The widening confidence intervals in the analysis of odds ratios for disease occurrence across genders clearly indicate that the diminishing sample sizes impact the result reliability. Thirdly, our analyses rely exclusively on ICD codes, which are known to be often incomplete, occasionally inaccurate, and influenced by differences in coding practices. Such inherent limitations of administrative coding data may introduce misclassification and potentially impact the accuracy of our findings. Fourth, by requiring prior hospital visits and adjusting diagnosis dates through clinical note review, our study may have preferentially included patients more engaged with healthcare services, introducing selection bias toward individuals with a higher comorbidity burden and potentially limiting the generalizability of our findings. Fifth, our mortality data are limited to in-hospital deaths recorded at the Clinical Center of the University of Debrecen; as a result, deaths occurring outside this institution (e.g., at home or in other facilities) were not captured, which may lead to underestimation of overall mortality rates and potentially influence interpretations of comorbidity trajectories related to survival. Lastly, with respect to the methods, it is important to note that lift can overestimate the influence of rare items in association rule mining.

## Conclusions

This study successfully utilized the Association Rule Mining (ARM) technique to reveal patterns of comorbidities across various timeframes surrounding the diagnosis of T2DM, explicitly accounting for imbalanced data. Our findings highlight the dense occurrence of new diagnosis particularly in the initial five years after the T2DM diagnosis, with conditions such as hypertension showing high prevalence. Renal- and respiratory system related complications show steady occurrence during the course of the disease after the diagnosis. The gender-specific analysis revealed notable differences in disease patterns, where certain conditions were mainly associated with females in the years leading up to the T2DM diagnosis. Additionally, the study highlighted the challenges associated with using electronic health records as a data source. Careful data preprocessing was necessary, along with the selection of appropriate techniques to address data imbalance. The data might be biased, considering it was collected by an institute that provides secondary healthcare. The insights gained emphasize the importance of early intervention and adaptive healthcare approaches for managing T2DM and its associated comorbidities.

## Electronic supplementary material

Below is the link to the electronic supplementary material.

Supplementary Material 1

## Data Availability

The data that support the findings of this study are available from Clinical Centre of the University of Debrecen, but restrictions apply to the availability of these data, which were used under license for the current study, and so are not publicly available. Data are however available from the authors upon reasonable request and with permission of Clinical Centre of the University of Debrecen, Hungary.
