# Supplementary material for: Association rule mining of time-based patterns in diabetes-related comorbidities on imbalanced data: a pre- and post-diagnosis study
Source: BMC Med Inform Decis Mak. 2025 Sep 29;25:352. doi: 10.1186/s12911-025-03206-1 (PMC12482723; doi:10.1186/s12911-025-03206-1)
Supplement: Supplementary file 1 — Supplementary Material 1 [file 12911_2025_3206_MOESM1_ESM.docx]

**Association Rule Mining of Time-Based Patterns in Diabetes-Related Comorbidities on Imbalanced Data: A Pre- and Post-Diagnosis Study**

Róbert Bata^1^, Amr Sayed Ghanem^1^, Vargáné Dr. Faludi Eszter^2^, Ferenc Sztanek^3^, Attila Csaba Nagy^1,*^

^1^Department of Health Informatics, Faculty of Health Sciences, University of Debrecen, Debrecen, Hungary

^2^Department of Integrative Health Sciences, Faculty of Health Sciences, University of Debrecen, Debrecen, Hungary

^3^Division of Metabolic Diseases, Department of Internal Medicine, University of Debrecen Faculty of Medicine, Debrecen, Hungary

^*^corresponding: nagy.attila@etk.unideb.hu

| 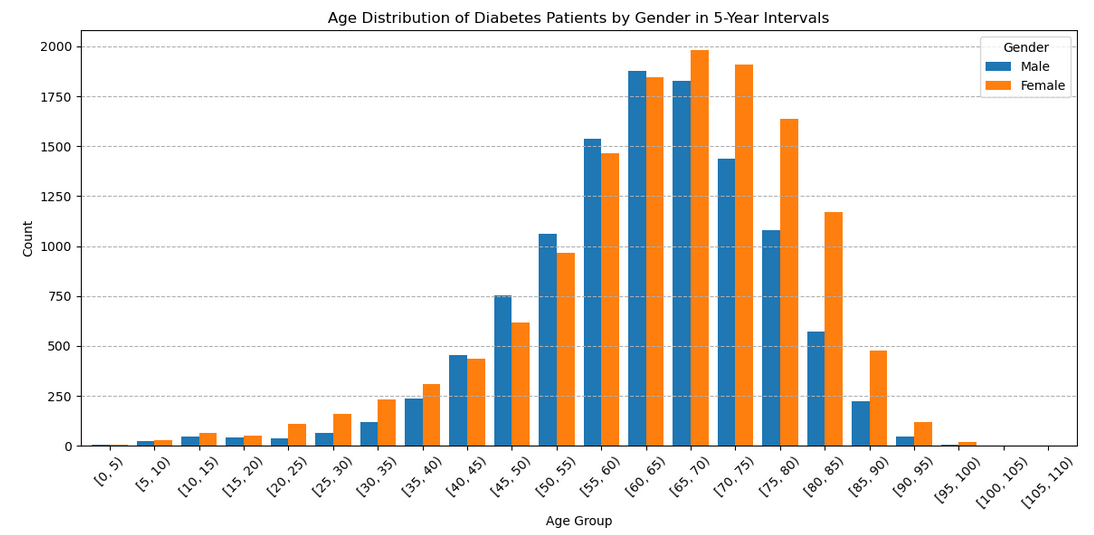  Supplementary Figure 1. Age distribution  Age distribution of T2DM patients in the study database. |
| --- |

| PATIENT_ID | DIAG_DT | ICD_SET_O | TD_FD | ICD_SET_U |
| --- | --- | --- | --- | --- |
| 981 | 2007 | N72, N60, N64, N87, N91 | -6 | N72, N64, N91, N87, N60 |
| 981 | 2008 | N60, N95, N92, N87, N91 | -5 | N92, N95 |
| 981 | 2010 | N76, N95, N87 | -3 | N76 |
| 981 | 2013 | N95, M14, G63, L85, E11, I79, L98 | 0 | I79, M14, E11, L98, L85, G63 |
| 981 | 2014 | N60, N95, N30, M14, G63, M81, N87, L85, E11, E05, I79, M51, L98 | 1 | E05, N30, M81, M51 |
| 981 | 2015 | N95, M14, I79, G63, L85, E11, I10, B97, A63, N76, N90, L98 | 2 | B97, N90, A63, I10 |
| 981 | 2016 | N95, N30, M14, G63, L85, E11, L97, I79, L98 | 3 | L97 |
| 981 | 2017 | N95, E06, M14, G63, N92, L85, E11, L97, B97, I79, L98 | 4 | E06 |
| 981 | 2020 | N95, N87, M65, B97, L98 | 7 | M65 |
| 981 | 2021 | M14, G63, L85, E11, L97, I79, M51, L98 | 8 |  |

Supplementary Table 1. Partial dataset

PATIENT_ID – Recoded patient ID, not the original; DIAG_DT - year of hospital visit when the patient was diagnosed; ICD_SET_O - original ICD code occurrences in the database; ICD_SET_U - ICD code occurrences after keeping just the first occurrence of the ICD codes; TD_FD – the time passed since the diagnosis of T2DM (E11)

| condition | timeframe | l.supp. | lift | excl.lift | OR (f) [95% CI] |
| --- | --- | --- | --- | --- | --- |
| Osteoporosis | n5-n1 | 0.102 | 1.612 | 0.58 | 0.20 [0.18–0.23] |
| Upper respiratory infection | n5-n1 | 0.036 | 1.605 | 0.592 | 0.66 [0.57–0.75] |
| Excessive, frequent and irregular menstruation | n5-n1 | 0.033 | 1.528 | 0.569 | 0.00 [0.00–0.00] |
| Spondylosis | n5-n1 | 0.138 | 1.492 | 0.549 | 0.79 [0.73–0.85] |
| Other soft tissue disorders, not elsewhere classified | n5-n1 | 0.091 | 1.472 | 0.452 | 0.80 [0.74–0.88] |
| Dorsalgia | n5-n1 | 0.119 | 1.449 | 0.461 | 0.83 [0.77–0.90] |
| Menopausal and other perimenopausal disorders | n5-n1 | 0.071 | 1.421 | 0.512 | 0.00 [0.00–0.00] |
| Other enthesopathies | n5-n1 | 0.044 | 1.405 | 0.508 | 0.67 [0.59–0.76] |
| Benign mammary dysplasia | n5-n1 | 0.063 | 1.364 | 0.468 | 0.05 [0.04–0.06] |
| Polyarthrosis | n5-n1 | 0.041 | 1.359 | 0.495 | 0.50 [0.44–0.57] |
| Other inflammation of vagina and vulva | n5-n1 | 0.033 | 1.347 | 0.496 | 0.00 [0.00–0.00] |
| Disorders of refraction and accommodation | n5-n1 | 0.187 | 1.333 | 0.496 | 0.78 [0.73–0.83] |
| Other intervertebral disc disorders | n5-n1 | 0.096 | 1.327 | 0.408 | 0.86 [0.79–0.93] |
| Other dorsopathies, not elsewhere classified | n5-n1 | 0.042 | 1.322 | 0.469 | 0.59 [0.51–0.67] |
| Calculus of kidney and ureter | n5-n1 | 0.046 | 1.314 | 0.468 | 1.05 [0.94–1.19] |
| Gonarthrosis [arthrosis of knee] | n5-n1 | 0.098 | 1.311 | 0.403 | 0.65 [0.60–0.71] |
| Conjunctivitis | n5-n1 | 0.075 | 1.309 | 0.479 | 0.75 [0.68–0.83] |
| Other joint disorders, not elsewhere classified | n5-n1 | 0.094 | 1.296 | 0.373 | 0.72 [0.66–0.79] |
| Vasomotor and allergic rhinitis | n5-n1 | 0.042 | 1.276 | 0.456 | 0.63 [0.56–0.72] |
| Acute pharyngitis | n5-n1 | 0.036 | 1.254 | 0.454 | 0.56 [0.49–0.65] |
| Coxarthrosis [arthrosis of hip] | n5-n1 | 0.07 | 1.249 | 0.38 | 0.64 [0.58–0.70] |
| Depressive episode | n5-n1 | 0.049 | 1.238 | 0.436 | 0.47 [0.41–0.53] |
| Shoulder lesions | n5-n1 | 0.054 | 1.233 | 0.425 | 0.95 [0.85–1.06] |
| Varicose veins of lower extremities | n5-n1 | 0.039 | 1.219 | 0.434 | 0.55 [0.48–0.63] |
| Diabetic retinopathy | n5-n1 | 0.147 | 1.218 | 0.414 | 0.84 [0.79–0.91] |
| Other nontoxic goitre | n5-n1 | 0.053 | 1.212 | 0.435 | 0.24 [0.21–0.28] |
| Otitis externa | n5-n1 | 0.051 | 1.205 | 0.425 | 0.99 [0.88–1.10] |
| Other cataract | n5-n1 | 0.045 | 1.171 | 0.4 | 0.70 [0.62–0.79] |
| Cholelithiasis | n5-n1 | 0.076 | 1.169 | 0.368 | 0.56 [0.51–0.62] |
| Other benign neoplasms of skin | n5-n1 | 0.057 | 1.16 | 0.408 | 0.79 [0.71–0.88] |
| Other disorders of external ear | n5-n1 | 0.074 | 1.158 | 0.411 | 1.08 [0.98–1.19] |
| Hypertension | n5-n1 | 0.305 | 1.145 | 0.407 | 0.98 [0.93–1.04] |
| Angina pectoris | n5-n1 | 0.152 | 1.124 | 0.37 | 0.99 [0.93–1.07] |
| Disorders of lacrimal system | n5-n1 | 0.042 | 1.117 | 0.407 | 0.40 [0.35–0.46] |
| Other acquired deformities of limbs | n5-n1 | 0.059 | 1.117 | 0.401 | 0.49 [0.44–0.55] |
| Haemorrhoids | n5-n1 | 0.042 | 1.117 | 0.385 | 0.96 [0.85–1.09] |
| Conductive and sensorineural hearing loss | n5-n1 | 0.044 | 1.11 | 0.386 | 1.19 [1.06–1.35] |
| Other anxiety disorders | n5-n1 | 0.068 | 1.098 | 0.385 | 0.49 [0.44–0.54] |
| Hypertensive heart disease | n5-n1 | 0.068 | 1.093 | 0.39 | 0.86 [0.78–0.95] |
| Hyperplasia of prostate | n5-n1 | 0.054 | 1.081 | 0.387 | 452.21 [169.42–1207.06] |
| Acquired deformities of fingers and toes | n5-n1 | 0.032 | 1.06 | 0.374 | 0.29 [0.24–0.34] |
| Postprocedural disorders of eye and adnexa, not elsewhere classified | n5-n1 | 0.05 | 1.057 | 0.287 | 0.76 [0.67–0.85] |
| Gastritis and duodenitis | n5-n1 | 0.061 | 1.025 | 0.286 | 0.79 [0.71–0.87] |
| Phlebitis and thrombophlebitis | n5-n1 | 0.033 | 1.023 | 0.346 | 1.01 [0.88–1.16] |
| Other dermatitis | n5-n1 | 0.061 | 1.017 | 0.304 | 0.75 [0.68–0.83] |
| Asthma | n5-n1 | 0.038 | 1.011 | 0.348 | 0.60 [0.53–0.69] |
| Other diseases of liver | n5-n1 | 0.06 | 0.987 | 0.349 | 1.28 [1.15–1.42] |
| Other arthrosis | n5-n1 | 0.04 | 0.986 | 0.339 | 0.64 [0.56–0.73] |
| Senile cataract | n5-n1 | 0.094 | 0.968 | 0.273 | 0.73 [0.67–0.80] |
| Ischemic heart disease | n5-n1 | 0.127 | 0.963 | 0.298 | 1.01 [0.93–1.09] |
| Thyrotoxicosis [hyperthyroidism] | n5-n1 | 0.058 | 0.948 | 0.256 | 0.22 [0.19–0.25] |
| Cerebral infarction | n5-n1 | 0.063 | 0.92 | 0.251 | 1.00 [0.91–1.11] |
| Cardiomyopathy | n5-n1 | 0.053 | 0.917 | 0.285 | 1.24 [1.11–1.39] |
| Other disorders of eye and adnexa | n5-n1 | 0.08 | 0.906 | 0.242 | 0.93 [0.85–1.02] |
| Obesity | n5-n1 | 0.084 | 0.885 | 0.327 | 0.66 [0.60–0.73] |
| Pneumonia | n5-n1 | 0.085 | 0.882 | 0.217 | 0.99 [0.91–1.08] |
| Other hypothyroidism | n5-n1 | 0.033 | 0.864 | 0.302 | 0.24 [0.20–0.28] |
| Other cardiac arrhythmias | n5-n1 | 0.044 | 0.84 | 0.237 | 1.14 [1.01–1.29] |
| Other chronic obstructive pulmonary disease | n5-n1 | 0.04 | 0.818 | 0.232 | 1.21 [1.07–1.37] |
| Acute bronchitis | n5-n1 | 0.049 | 0.816 | 0.207 | 0.81 [0.72–0.91] |
| Transient cerebral ischaemic attacks and related syndromes | n5-n1 | 0.031 | 0.814 | 0.213 | 0.79 [0.68–0.91] |
| Hyperlipidemia | n5-n1 | 0.124 | 0.808 | 0.267 | 0.93 [0.86–1.00] |
| Other disorders of thyroid | n5-n1 | 0.033 | 0.804 | 0.227 | 0.23 [0.20–0.28] |
| Atherosclerosis | n5-n1 | 0.065 | 0.795 | 0.222 | 1.20 [1.09–1.33] |
| reflux disease | n5-n1 | 0.048 | 0.791 | 0.214 | 0.76 [0.68–0.86] |
| Other cerebrovascular diseases | n5-n1 | 0.049 | 0.787 | 0.237 | 0.66 [0.58–0.74] |
| Nonrheumatic mitral valve disorders | n5-n1 | 0.032 | 0.697 | 0.192 | 0.70 [0.61–0.81] |
| Lower urinary infection | n5-n1 | 0.031 | 0.693 | 0.165 | 0.32 [0.27–0.38] |
| Cystitis | n5-n1 | 0.046 | 0.68 | 0.168 | 0.32 [0.28–0.37] |
| Occlusion and stenosis of precerebral arteries, not resulting in cerebral infarction | n5-n1 | 0.03 | 0.628 | 0.158 | 1.38 [1.19–1.59] |
| Chronic heart failure | n5-n1 | 0.055 | 0.62 | 0.158 | 1.09 [0.98–1.22] |
| Atrial fibrillation and flutter | n5-n1 | 0.038 | 0.618 | 0.158 | 1.20 [1.06–1.37] |
| Polyneuropathy in diseases classified elsewhere | 0-4 | 0.076 | 1.766 | 0.452 | 0.99 [0.90–1.09] |
| Hyperuricemia | 0-4 | 0.06 | 1.581 | 0.435 | 0.91 [0.82–1.01] |
| Chronic respiratory failure | 0-4 | 0.074 | 1.542 | 0.314 | 1.48 [1.35–1.63] |
| Other disorders of fluid, electrolyte and acid-base balance | 0-4 | 0.069 | 1.54 | 0.344 | 1.12 [1.02–1.24] |
| Other respiratory disorders | 0-4 | 0.039 | 1.54 | 0.329 | 1.37 [1.20–1.56] |
| Chronic kidney disease | 0-4 | 0.116 | 1.53 | 0.35 | 0.90 [0.83–0.97] |
| Acute kidney injury | 0-4 | 0.044 | 1.505 | 0.301 | 1.26 [1.12–1.43] |
| Volume depletion | 0-4 | 0.052 | 1.488 | 0.311 | 0.82 [0.73–0.92] |
| Other anaemias | 0-4 | 0.094 | 1.486 | 0.351 | 0.93 [0.85–1.01] |
| Rheumatic tricuspid valve diseases | 0-4 | 0.047 | 1.472 | 0.388 | 0.90 [0.80–1.02] |
| Hyperlipidemia | 0-4 | 0.225 | 1.466 | 0.485 | 1.05 [0.99–1.11] |
| Obesity | 0-4 | 0.136 | 1.435 | 0.53 | 0.85 [0.79–0.91] |
| Chronic heart failure | 0-4 | 0.124 | 1.412 | 0.36 | 1.19 [1.10–1.28] |
| Atrioventricular and left bundle-branch block | 0-4 | 0.031 | 1.405 | 0.466 | 1.48 [1.28–1.71] |
| Nonrheumatic aortic valve disorders | 0-4 | 0.042 | 1.401 | 0.368 | 0.99 [0.87–1.12] |
| Atrial fibrillation and flutter | 0-4 | 0.085 | 1.396 | 0.356 | 1.22 [1.12–1.34] |
| Nonrheumatic mitral valve disorders | 0-4 | 0.062 | 1.377 | 0.38 | 0.79 [0.71–0.87] |
| Other cerebrovascular diseases | 0-4 | 0.086 | 1.375 | 0.414 | 0.70 [0.64–0.77] |
| Occlusion and stenosis of precerebral arteries, not resulting in cerebral infarction | 0-4 | 0.065 | 1.35 | 0.339 | 1.39 [1.25–1.53] |
| Atherosclerosis | 0-4 | 0.108 | 1.325 | 0.371 | 1.11 [1.02–1.20] |
| Peripheral arterial disease | 0-4 | 0.032 | 1.324 | 0.299 | 2.00 [1.73–2.32] |
| Other hypothyroidism | 0-4 | 0.051 | 1.315 | 0.46 | 0.28 [0.24–0.32] |
| Cardiomyopathy | 0-4 | 0.075 | 1.308 | 0.406 | 1.41 [1.29–1.55] |
| Other disorders of thyroid | 0-4 | 0.053 | 1.299 | 0.366 | 0.30 [0.27–0.35] |
| Other disorders of veins | 0-4 | 0.032 | 1.298 | 0.432 | 0.72 [0.62–0.83] |
| Other mental disorders due to brain damage and dysfunction and to physical disease | 0-4 | 0.044 | 1.281 | 0.315 | 0.80 [0.71–0.91] |
| reflux disease | 0-4 | 0.078 | 1.28 | 0.347 | 0.87 [0.79–0.96] |
| Other chronic obstructive pulmonary disease | 0-4 | 0.063 | 1.28 | 0.363 | 1.44 [1.30–1.59] |
| Other cardiac arrhythmias | 0-4 | 0.067 | 1.274 | 0.359 | 1.09 [0.99–1.21] |
| Cystitis | 0-4 | 0.086 | 1.265 | 0.312 | 0.49 [0.45–0.54] |
| Lower urinary infection | 0-4 | 0.057 | 1.263 | 0.302 | 0.59 [0.52–0.66] |
| Ischemic heart disease | 0-4 | 0.165 | 1.256 | 0.388 | 1.19 [1.11–1.27] |
| Transient cerebral ischaemic attacks and related syndromes | 0-4 | 0.047 | 1.233 | 0.323 | 1.05 [0.93–1.18] |
| Benign neoplasm of colon, rectum, anus and anal canal | 0-4 | 0.031 | 1.232 | 0.396 | 1.37 [1.18–1.58] |
| Other local infections of skin and subcutaneous tissue | 0-4 | 0.03 | 1.228 | 0.303 | 1.21 [1.04–1.40] |
| Other polyneuropathies | 0-4 | 0.049 | 1.225 | 0.288 | 1.07 [0.95–1.20] |
| Other diseases of liver | 0-4 | 0.074 | 1.219 | 0.43 | 1.13 [1.03–1.24] |
| Hypertension | 0-4 | 0.324 | 1.217 | 0.433 | 1.14 [1.08–1.20] |
| Acquired deformities of fingers and toes | 0-4 | 0.036 | 1.187 | 0.419 | 0.28 [0.24–0.34] |
| Acute bronchitis | 0-4 | 0.071 | 1.179 | 0.299 | 1.03 [0.93–1.13] |
| Disorders of lacrimal system | 0-4 | 0.044 | 1.178 | 0.428 | 0.44 [0.38–0.50] |
| Other arthrosis | 0-4 | 0.047 | 1.163 | 0.4 | 0.56 [0.49–0.63] |
| Other acquired deformities of limbs | 0-4 | 0.061 | 1.159 | 0.416 | 0.46 [0.41–0.52] |
| Acute viral infection | 0-4 | 0.045 | 1.154 | 0.199 | 0.83 [0.74–0.94] |
| Cerebral infarction | 0-4 | 0.079 | 1.153 | 0.314 | 1.09 [0.99–1.19] |
| Hyperplasia of prostate | 0-4 | 0.057 | 1.15 | 0.412 | 1932.61 [271.99–13732.04] |
| Asthma | 0-4 | 0.043 | 1.148 | 0.395 | 0.68 [0.60–0.77] |
| Other dermatitis | 0-4 | 0.069 | 1.147 | 0.343 | 0.78 [0.70–0.86] |
| Angina pectoris | 0-4 | 0.154 | 1.14 | 0.375 | 0.98 [0.91–1.05] |
| Senile cataract | 0-4 | 0.11 | 1.134 | 0.319 | 0.70 [0.64–0.76] |
| Pneumonia | 0-4 | 0.109 | 1.133 | 0.279 | 1.21 [1.12–1.31] |
| Seborrhoeic keratosis | 0-4 | 0.035 | 1.129 | 0.375 | 0.60 [0.52–0.70] |
| Hypertensive heart disease | 0-4 | 0.07 | 1.129 | 0.403 | 1.06 [0.96–1.17] |
| Other disorders of eye and adnexa | 0-4 | 0.098 | 1.112 | 0.297 | 0.91 [0.84–0.99] |
| Other anxiety disorders | 0-4 | 0.069 | 1.109 | 0.388 | 0.58 [0.52–0.64] |
| Gastritis and duodenitis | 0-4 | 0.065 | 1.084 | 0.303 | 0.91 [0.82–1.01] |
| Thyrotoxicosis [hyperthyroidism] | 0-4 | 0.066 | 1.079 | 0.292 | 0.23 [0.20–0.26] |
| Diabetic retinopathy | 0-4 | 0.129 | 1.074 | 0.366 | 0.86 [0.80–0.92] |
| Other disorders of external ear | 0-4 | 0.068 | 1.07 | 0.38 | 0.92 [0.83–1.02] |
| Cholelithiasis | 0-4 | 0.07 | 1.064 | 0.335 | 0.66 [0.59–0.73] |
| Conductive and sensorineural hearing loss | 0-4 | 0.042 | 1.061 | 0.369 | 0.89 [0.79–1.01] |
| Phlebitis and thrombophlebitis | 0-4 | 0.034 | 1.052 | 0.356 | 0.98 [0.86–1.13] |
| Other benign neoplasms of skin | 0-4 | 0.052 | 1.05 | 0.369 | 0.68 [0.60–0.76] |
| Other nontoxic goitre | 0-4 | 0.046 | 1.044 | 0.375 | 0.30 [0.26–0.35] |
| Acute pharyngitis | 0-4 | 0.03 | 1.032 | 0.374 | 0.62 [0.53–0.72] |
| Otitis externa | 0-4 | 0.043 | 1.016 | 0.359 | 0.93 [0.82–1.06] |
| Postprocedural disorders of eye and adnexa, not elsewhere classified | 0-4 | 0.047 | 1.009 | 0.274 | 0.82 [0.73–0.92] |
| Varicose veins of lower extremities | 0-4 | 0.032 | 1.009 | 0.36 | 0.64 [0.55–0.74] |
| Haemorrhoids | 0-4 | 0.038 | 1.005 | 0.347 | 0.97 [0.85–1.10] |
| Conjunctivitis | 0-4 | 0.058 | 1.002 | 0.367 | 0.71 [0.63–0.79] |
| Disorders of refraction and accommodation | 0-4 | 0.14 | 0.998 | 0.371 | 0.83 [0.77–0.89] |
| Depressive episode | 0-4 | 0.039 | 0.974 | 0.343 | 0.60 [0.52–0.68] |
| Other cataract | 0-4 | 0.037 | 0.966 | 0.33 | 0.65 [0.57–0.75] |
| Vasomotor and allergic rhinitis | 0-4 | 0.032 | 0.965 | 0.345 | 0.66 [0.57–0.77] |
| Coxarthrosis [arthrosis of hip] | 0-4 | 0.053 | 0.938 | 0.285 | 0.72 [0.64–0.81] |
| Shoulder lesions | 0-4 | 0.04 | 0.917 | 0.316 | 0.95 [0.84–1.08] |
| Calculus of kidney and ureter | 0-4 | 0.032 | 0.912 | 0.325 | 1.17 [1.01–1.34] |
| Gonarthrosis [arthrosis of knee] | 0-4 | 0.067 | 0.891 | 0.274 | 0.73 [0.66–0.80] |
| Other intervertebral disc disorders | 0-4 | 0.063 | 0.864 | 0.266 | 0.93 [0.84–1.03] |
| Other joint disorders, not elsewhere classified | 0-4 | 0.061 | 0.84 | 0.241 | 0.79 [0.71–0.88] |
| Menopausal and other perimenopausal disorders | 0-4 | 0.041 | 0.831 | 0.3 | 0.00 [0.00–0.00] |
| Spondylosis | 0-4 | 0.075 | 0.805 | 0.296 | 0.90 [0.82–0.99] |
| Dorsalgia | 0-4 | 0.065 | 0.784 | 0.249 | 0.89 [0.81–0.99] |
| Benign mammary dysplasia | 0-4 | 0.036 | 0.777 | 0.267 | 0.09 [0.07–0.11] |
| Other soft tissue disorders, not elsewhere classified | 0-4 | 0.046 | 0.737 | 0.227 | 0.91 [0.81–1.02] |
| Osteoporosis | 0-4 | 0.04 | 0.629 | 0.226 | 0.34 [0.29–0.39] |
| Acute viral infection | 5-9 | 0.062 | 1.57 | 0.271 | 1.00 [0.84–1.18] |
| Acute kidney injury | 5-9 | 0.045 | 1.527 | 0.306 | 1.10 [0.91–1.34] |
| Peripheral arterial disease | 5-9 | 0.036 | 1.467 | 0.332 | 2.05 [1.64–2.55] |
| Volume depletion | 5-9 | 0.05 | 1.431 | 0.299 | 0.88 [0.73–1.06] |
| Chronic respiratory failure | 5-9 | 0.069 | 1.431 | 0.291 | 1.25 [1.07–1.47] |
| Other disorders of bone density and structure | 5-9 | 0.033 | 1.413 | 0.433 | 0.30 [0.22–0.40] |
| Other respiratory disorders | 5-9 | 0.035 | 1.389 | 0.297 | 1.33 [1.07–1.66] |
| Other disorders of fluid, electrolyte and acid-base balance | 5-9 | 0.061 | 1.353 | 0.302 | 1.07 [0.90–1.27] |
| Other polyneuropathies | 5-9 | 0.052 | 1.281 | 0.301 | 1.16 [0.97–1.39] |
| Chronic kidney disease | 5-9 | 0.095 | 1.252 | 0.287 | 0.86 [0.75–0.99] |
| Other mental disorders | 5-9 | 0.042 | 1.222 | 0.301 | 0.70 [0.57–0.86] |
| Diverticulosis of large intestine | 5-9 | 0.032 | 1.167 | 0.374 | 0.92 [0.73–1.17] |
| Cystitis | 5-9 | 0.08 | 1.165 | 0.287 | 0.79 [0.68–0.92] |
| Polyneuropathy in diseases classified elsewhere | 5-9 | 0.049 | 1.142 | 0.293 | 1.09 [0.91–1.32] |
| Occlusion and stenosis of precerebral arteries, not resulting in cerebral infarction | 5-9 | 0.052 | 1.085 | 0.272 | 1.44 [1.20–1.73] |
| Other anaemias | 5-9 | 0.068 | 1.082 | 0.255 | 1.09 [0.93–1.29] |
| Lower urinary infection | 5-9 | 0.049 | 1.082 | 0.258 | 0.76 [0.62–0.92] |
| Acute bronchitis | 5-9 | 0.062 | 1.043 | 0.265 | 1.06 [0.90–1.26] |
| Other disorders of eye and adnexa | 5-9 | 0.092 | 1.04 | 0.278 | 1.14 [0.99–1.32] |
| Thyrotoxicosis [hyperthyroidism] | 5-9 | 0.063 | 1.027 | 0.278 | 0.29 [0.23–0.35] |
| Atrial fibrillation and flutter | 5-9 | 0.061 | 0.993 | 0.253 | 1.35 [1.14–1.60] |
| Rheumatic tricuspid valve diseases | 5-9 | 0.031 | 0.99 | 0.261 | 0.98 [0.78–1.24] |
| Chronic heart failure | 5-9 | 0.082 | 0.935 | 0.239 | 1.29 [1.11–1.49] |
| Pneumonia | 5-9 | 0.09 | 0.935 | 0.23 | 1.29 [1.12–1.49] |
| Transient cerebral ischaemic attacks and related syndromes | 5-9 | 0.035 | 0.925 | 0.243 | 1.03 [0.83–1.29] |
| Hyperuricemia | 5-9 | 0.035 | 0.91 | 0.251 | 0.95 [0.76–1.19] |
| Nonrheumatic mitral valve disorders | 5-9 | 0.041 | 0.908 | 0.251 | 1.01 [0.82–1.23] |
| Postprocedural disorders of eye and adnexa, not elsewhere classified | 5-9 | 0.042 | 0.9 | 0.244 | 0.89 [0.72–1.09] |
| Cerebral infarction | 5-9 | 0.061 | 0.887 | 0.242 | 1.08 [0.91–1.27] |
| reflux disease | 5-9 | 0.054 | 0.886 | 0.24 | 0.96 [0.80–1.15] |
| Other chronic obstructive pulmonary disease | 5-9 | 0.043 | 0.862 | 0.244 | 1.25 [1.03–1.53] |
| Other disorders of thyroid | 5-9 | 0.034 | 0.837 | 0.236 | 0.27 [0.20–0.36] |
| Senile cataract | 5-9 | 0.081 | 0.834 | 0.235 | 0.84 [0.73–0.98] |
| Gastritis and duodenitis | 5-9 | 0.048 | 0.804 | 0.225 | 0.92 [0.76–1.11] |
| Other cardiac arrhythmias | 5-9 | 0.042 | 0.801 | 0.226 | 1.11 [0.91–1.36] |
| Other cataract | 5-9 | 0.031 | 0.789 | 0.27 | 0.73 [0.57–0.93] |
| Atherosclerosis | 5-9 | 0.063 | 0.775 | 0.217 | 1.24 [1.05–1.47] |
| Benign mammary dysplasia | 5-9 | 0.036 | 0.77 | 0.265 | 0.07 [0.05–0.12] |
| Other arthrosis | 5-9 | 0.031 | 0.76 | 0.261 | 0.71 [0.56–0.91] |
| Shoulder lesions | 5-9 | 0.033 | 0.748 | 0.258 | 1.11 [0.88–1.39] |
| Other joint disorders, not elsewhere classified | 5-9 | 0.054 | 0.745 | 0.214 | 1.04 [0.87–1.24] |
| Other cerebrovascular diseases | 5-9 | 0.045 | 0.73 | 0.22 | 0.98 [0.80–1.19] |
| Other dermatitis | 5-9 | 0.043 | 0.708 | 0.211 | 0.91 [0.74–1.11] |
| Coxarthrosis [arthrosis of hip] | 5-9 | 0.037 | 0.651 | 0.198 | 0.85 [0.68–1.06] |
| Other intervertebral disc disorders | 5-9 | 0.047 | 0.649 | 0.2 | 1.02 [0.84–1.24] |
| Other anxiety disorders | 5-9 | 0.04 | 0.647 | 0.227 | 0.60 [0.48–0.75] |
| Other benign neoplasms of skin | 5-9 | 0.031 | 0.631 | 0.222 | 0.70 [0.55–0.90] |
| Other diseases of liver | 5-9 | 0.038 | 0.627 | 0.221 | 1.22 [0.99–1.51] |
| Gonarthrosis [arthrosis of knee] | 5-9 | 0.046 | 0.617 | 0.19 | 0.93 [0.77–1.13] |
| Other disorders of external ear | 5-9 | 0.038 | 0.589 | 0.209 | 1.06 [0.86–1.31] |
| Other soft tissue disorders, not elsewhere classified | 5-9 | 0.036 | 0.588 | 0.181 | 1.27 [1.03–1.58] |
| Hypertensive heart disease | 5-9 | 0.036 | 0.578 | 0.206 | 1.35 [1.09–1.68] |
| Ischemic heart disease | 5-9 | 0.076 | 0.576 | 0.178 | 1.32 [1.14–1.54] |
| Cardiomyopathy | 5-9 | 0.033 | 0.565 | 0.176 | 1.86 [1.48–2.34] |
| Dorsalgia | 5-9 | 0.045 | 0.549 | 0.175 | 1.09 [0.90–1.32] |
| Cholelithiasis | 5-9 | 0.036 | 0.546 | 0.172 | 0.77 [0.61–0.96] |
| Osteoporosis | 5-9 | 0.034 | 0.537 | 0.193 | 0.45 [0.35–0.58] |
| Angina pectoris | 5-9 | 0.067 | 0.498 | 0.164 | 1.23 [1.05–1.45] |
| Hyperlipidemia | 5-9 | 0.074 | 0.479 | 0.159 | 1.04 [0.89–1.22] |
| Diabetic retinopathy | 5-9 | 0.053 | 0.442 | 0.15 | 0.89 [0.74–1.07] |
| Spondylosis | 5-9 | 0.039 | 0.421 | 0.155 | 1.06 [0.86–1.31] |
| Obesity | 5-9 | 0.037 | 0.385 | 0.142 | 0.85 [0.68–1.06] |
| Disorders of refraction and accommodation | 5-9 | 0.05 | 0.359 | 0.133 | 1.01 [0.84–1.21] |
| Hypertension | 5-9 | 0.071 | 0.266 | 0.094 | 1.21 [1.03–1.42] |
| Acute viral infection | 10- | 0.102 | 2.6 | 0.449 | 0.99 [0.76–1.28] |
| Unspecified kidney failure | 10- | 0.035 | 2.427 | 0.437 | 1.45 [0.95–2.22] |
| Acute kidney injury | 10- | 0.051 | 1.737 | 0.348 | 1.64 [1.15–2.34] |
| Chronic respiratory failure | 10- | 0.083 | 1.715 | 0.349 | 1.44 [1.08–1.91] |
| Volume depletion | 10- | 0.054 | 1.569 | 0.328 | 1.10 [0.78–1.56] |
| Other respiratory disorders | 10- | 0.037 | 1.487 | 0.318 | 1.01 [0.67–1.54] |
| Other diseases of digestive system | 10- | 0.031 | 1.45 | 0.319 | 1.21 [0.77–1.90] |
| Other disorders of fluid, electrolyte and acid-base balance | 10- | 0.057 | 1.277 | 0.285 | 1.35 [0.97–1.89] |
| Chronic kidney disease | 10- | 0.094 | 1.232 | 0.282 | 1.11 [0.84–1.45] |
| Other anaemias | 10- | 0.076 | 1.202 | 0.284 | 0.98 [0.73–1.33] |
| Lower urinary infection | 10- | 0.052 | 1.151 | 0.275 | 1.51 [1.06–2.14] |
| Pneumonia | 10- | 0.107 | 1.106 | 0.273 | 1.25 [0.97–1.61] |
| Other polyneuropathies | 10- | 0.044 | 1.084 | 0.255 | 0.98 [0.67–1.44] |
| Chronic heart failure | 10- | 0.084 | 0.952 | 0.243 | 1.75 [1.32–2.32] |
| Cystitis | 10- | 0.065 | 0.948 | 0.234 | 1.06 [0.77–1.46] |
| Occlusion and stenosis of precerebral arteries, not resulting in cerebral infarction | 10- | 0.044 | 0.92 | 0.231 | 1.64 [1.12–2.41] |
| Other mental disorders due to brain damage and dysfunction and to physical disease | 10- | 0.031 | 0.917 | 0.226 | 0.93 [0.59–1.46] |
| Atrial fibrillation and flutter | 10- | 0.056 | 0.914 | 0.233 | 1.41 [1.01–1.98] |
| Acute bronchitis | 10- | 0.054 | 0.902 | 0.229 | 1.63 [1.15–2.30] |
| Transient cerebral ischaemic attacks and related syndromes | 10- | 0.032 | 0.84 | 0.22 | 1.45 [0.93–2.27] |
| Polyneuropathy in diseases classified elsewhere | 10- | 0.034 | 0.789 | 0.202 | 1.19 [0.78–1.84] |
| reflux disease | 10- | 0.045 | 0.737 | 0.199 | 1.41 [0.97–2.06] |
| Postprocedural disorders of eye and adnexa, not elsewhere classified | 10- | 0.034 | 0.717 | 0.195 | 1.22 [0.79–1.89] |
| Cerebral infarction | 10- | 0.048 | 0.708 | 0.193 | 1.21 [0.84–1.74] |
| Other disorders of eye and adnexa | 10- | 0.06 | 0.683 | 0.183 | 1.11 [0.80–1.55] |
| Atherosclerosis | 10- | 0.055 | 0.678 | 0.19 | 1.46 [1.03–2.05] |
| Gastritis and duodenitis | 10- | 0.04 | 0.664 | 0.186 | 0.87 [0.58–1.31] |
| Thyrotoxicosis [hyperthyroidism] | 10- | 0.039 | 0.642 | 0.174 | 0.31 [0.19–0.52] |
| Other cardiac arrhythmias | 10- | 0.033 | 0.63 | 0.178 | 1.35 [0.87–2.09] |
| Senile cataract | 10- | 0.06 | 0.615 | 0.173 | 1.14 [0.82–1.59] |
| Other joint disorders, not elsewhere classified | 10- | 0.043 | 0.598 | 0.172 | 0.75 [0.50–1.12] |
| Ischemic heart disease | 10- | 0.058 | 0.44 | 0.136 | 1.35 [0.97–1.89] |
| Gonarthrosis [arthrosis of knee] | 10- | 0.033 | 0.435 | 0.134 | 1.02 [0.65–1.59] |
| Angina pectoris | 10- | 0.037 | 0.276 | 0.091 | 1.45 [0.96–2.18] |
| Hyperlipidemia | 10- | 0.041 | 0.269 | 0.089 | 1.56 [1.05–2.31] |
| Hypertension | 10- | 0.049 | 0.185 | 0.066 | 1.70 [1.18–2.45] |

Supplementary Table 2. All the generated rules for the 0.03 threshold

This table presents the individual association rules identified across the four timeframes relative to T2DM diagnosis (n5–n1, 0–4, 5–9, 10+), using a minimum local support threshold of 0.03. Each rule includes the antecedent comorbidity, the timeframe as consequent, and associated metrics such as local support, lift, and exclusiveness lift, highlighting temporal patterns of comorbidity occurrence. Crude odds ratios (OR) with 95% confidence intervals (CI) are also provided, calculated with female as the reference category for gender comparisons.

| Condition | n5-n1 | 0-4 | 5-9 | 10- |
| --- | --- | --- | --- | --- |
| Hypertension | 1.628 | 1.731 | 0.378 | 0.263 |
| Hyperlipidaemia | 1.069 | 1.940 | 0.634 | 0.356 |
| Disorders of refraction and accommodation | 1.895 | 1.418 | 0.510 | 0.177 |
| Angina pectoris | 1.481 | 1.501 | 0.656 | 0.363 |
| Ischemic heart disease | 1.191 | 1.553 | 0.712 | 0.544 |
| Diabetic retinopathy | 1.658 | 1.462 | 0.602 | 0.278 |
| Senile cataract | 1.091 | 1.278 | 0.939 | 0.692 |
| Pneumonia | 0.870 | 1.118 | 0.922 | 1.091 |
| Obesity | 1.234 | 2.001 | 0.538 | 0.227 |
| Spondylosis | 2.035 | 1.099 | 0.574 | 0.292 |
| Other disorders of eye and adnexa | 0.969 | 1.189 | 1.112 | 0.731 |
| Chronic heart failure | 0.633 | 1.441 | 0.954 | 0.972 |
| Dorsalgia | 1.843 | 0.998 | 0.698 | 0.460 |
| Atherosclerosis | 0.890 | 1.483 | 0.868 | 0.759 |
| Chronic kidney disease | 0.325 | 1.401 | 1.146 | 1.128 |

Supplementary Table 3. Adjusted ratio

Adjusted ratio for the 15 most prevalent diseases across the four timeframes

| **Variable** | **Category** | **N(%)** |
| --- | --- | --- |
| **Age (all)** | Mean (SD) | 59.42 (14.36) |
|  | Median (IQR) | 61.00 (51.00-69.00) |
| **Age (male)** | Mean (SD) | 58.54 (13.39) |
|  | Median (IQR) | 59.00 (51.00-68.00) |
| **Age (female)** | Mean (SD) | 60.16 (15.09) |
|  | Median (IQR) | 62.00 (52.00-71.00) |
| **Gender** | female | 13606 (54.28%) |
|  | male | 11459 (45.72%) |
| **Hypertension** | yes | 5517 (22.01%) |
|  | no | 19548 (77.99 %) |
| **Disorders of refraction and accommodation** | yes | 3370 (13.45%) |
|  | no | 21695 (86.55 %) |
| **Angina pectoris** | yes | 2724 (10.87%) |
|  | no | 22341 (89.13 %) |
| **Diabetic retinopathy** | yes | 2614 (10.43%) |
|  | no | 22451 (89.57 %) |
| **Spondylosis** | yes | 2610 (10.41 %) |
|  | no | 22455 (89.59%) |

Supplementary Table 4. Baseline characteristics of the study cohort

Demographic and selected clinical characteristics at baseline (first entry) for all patients included in the analysis (N = 25,065). Age is presented as mean (standard deviation) and median (interquartile range), shown overall and stratified by gender. Frequencies and percentages are provided for gender distribution and selected comorbid conditions.

**Supplementary Methods: Association Rule Mining Metrics**

ARM can be represented as a rule in the form *A → c*, where *A* and *c* are disjoint item sets. *A* is the antecedent *c* is the consequent of the rule. With the help of various metrics, we quantify the dependence between the antecedent and the consequent. *Support* is the fraction of rules that contain both *A* and *c* may be regarded as the empirical joint probability. Typically, we are interested in the frequent rules, which support is higher than the user-defined minimum support. *Local support* is the ratio of the rules *(A → c)* in a certain timeframe *(c)*. *Confidence* of a rule is the conditional probability that defines how frequently the consequent *c* appears in transactions where there is antecedent *A*. *Lift* is the ratio of the observed joint probability of *A* and *c* to the expected joint probability if they were statistically independent. The lift value is indicating correlation between *A* and *c* by measuring how much the observed joint probability *P(Ac)* deviates from the independence assumption *P(A) * P(c).* We cannot heavily rely on the confidence measures since it can provide misleading rules. In case of an *A → c* association, the confidence metric only accounts for the importance of item *A* by heavily ignoring the relevance of item *c*, while the lift metric addresses this issue by measuring the strength of association between *A* and *c*. Having unbalanced classes, the relevance of *c*, the indirect class size, cannot be ignored, so *confidence* is not considered in the rule evaluation.
